# Comparing Tumor Cell Invasion and Myeloid Cell Composition in Compatible Primary and Relapsing Glioblastoma

**DOI:** 10.3390/cancers13143636

**Published:** 2021-07-20

**Authors:** Dongxu Zhao, Huabin Zhang, Ramazan Uyar, Jubayer A. Hossain, Hrvoje Miletic, Jörg-Christian Tonn, Rainer Glass, Roland E. Kälin

**Affiliations:** 1 Neurosurgical Research, University Hospital Ludwig Maximilian University Munich, 81377 Munich, Germany; Dongxu.Zhao@med.uni-muenchen.de (D.Z.); Huabin.Zhang@med.uni-muenchen.de (H.Z.); uyar.ramazan21@gmail.com (R.U.); 2 Walter Brendel Center of Experimental Medicine, Faculty of Medicine, Ludwig Maximilian University of Munich, 81377 Munich, Germany; 3 Department of Biomedicine, University of Bergen, 5021 Bergen, Norway; jubayer.hossain@uib.no (J.A.H.); Hrvoje.Miletic@uib.no (H.M.); 4 Department of Pathology, Haukeland University Hospital, 5021 Bergen, Norway; 5 Department of Neurosurgery, University Hospital Ludwig Maximilian University Munich, 81377 Munich, Germany; Joerg.Christian.Tonn@med.uni-muenchen.de; 6 German Cancer Research Center (DKFZ), German Cancer Consortium (DKTK), Partner Site Munich, 69120 Heidelberg, Germany

**Keywords:** recurrent glioblastoma, GBM relapse, HSV-thymidine kinase (HSVTK), ganciclovir (GCV), tumor associated myeloid cells (TAM), monocyte-derived macrophages (MDM), microglia, tumor cell invasion

## Abstract

**Simple Summary:**

We established a new minimally invasive mouse model for GBM relapse. For this, we utilized orthotopical implantation of HSVTK-transduced GBM cells and pharmacological treatment with GCV. In addition, we implanted patient-derived GBM cells of primary or recurrent tumors. We found that recurrent GBM were more aggressively invasive than primary GBM. Moreover, the recurring tumors had a higher ratio of monocyte-derived macrophages among the entire population of tumor associated myeloid cells. This shift in the composition of tumor-associated immune cells appeared to be independent from cell-death signaling or surgical intervention. This model provides the means to investigate the entire process of tumor relapse and test standard as well as experimental therapeutic strategies for relapsing GBM under defined conditions.

**Abstract:**

Glioblastoma (GBM) recurrence after treatment is almost inevitable but addressing this issue with adequate preclinical models has remained challenging. Here, we introduce a GBM mouse model allowing non-invasive and scalable de-bulking of a tumor mass located deeply in the brain, which can be combined with conventional therapeutic approaches. Strong reduction of the GBM volume is achieved after pharmacologically inducing a tumor-specific cell death mechanism. This is followed by GBM re-growth over a predictable timeframe. Pharmacological de-bulking followed by tumor relapse was accomplished with an orthotopic mouse glioma model. Relapsing experimental tumors recapitulated pathological features often observed in recurrent human GBM, like increased invasiveness or altered immune cell composition. Orthotopic implantation of GBM cells originating from biopsies of one patient at initial or follow-up treatment reproduced these findings. Interestingly, relapsing GBM of both models contained a much higher ratio of monocyte-derived macrophages (MDM) versus microglia than primary GBM. This was not altered when combining pharmacological de-bulking with invasive surgery. We interpret that factors released from viable primary GBM cells preferentially attract microglia whereas relapsing tumors preponderantly release chemoattractants for MDM. All in all, this relapse model has the capacity to provide novel insights into clinically highly relevant aspects of GBM treatment.

## 1. Introduction

Glioblastoma multiforme (GBM) is the most common and malignant tumor arising from the central nervous system (CNS) and prognosis for GBM patients remains very poor [1,2]. Patients diagnosed with GBM have a median survival of about 15 months after standard therapy consisting of tumor resection followed by a combination of radio- and chemotherapy [3,4]. GBM usually is fatal at first or second relapse. Despite many significant developments in the research and treatment of primary GBM, much less is known about the molecular characteristics of recurrent GBM [5,6,7]. Statistically, roughly two-thirds of recurrent GBM relapse within 2 cm of the primary tumor margin [8,9]. One-third of glioblastoma recur at distant sites, including different brain lobes or even infratentorial locations [10]. Owing to the tumor location or the impaired clinical condition of the patient microsurgical resection of the recurrent GBM is often not safely possible. This also negatively affects the advancement of research on the comparison of primary with recurrent tumor cells [11].

One additional constraint for developing new therapeutic options specifically against tumor recurrence is the paucity of adequate preclinical models. In particular, investigating the role of the tumor microenvironment (TME) in GBM relapse may indicate new therapeutic options, as the TME is less prone to therapy-induced hyper-mutation and acquired therapy-resistance than the GBM cells. The TME consists of a complex mix of brain resident cells, such as microglia, astrocytes, and neurons but also the neoplastic vasculature and peripherally invading immune cells (monocytes derived macrophages, abbreviated as MDM, and T-cells) [12,13,14]. Up to one third of the tumor mass is contributed by brain-resident microglia or peripherally invading MDM, which are commonly summarized as tumor-associated myeloid cells (TAM) [12,15]. These TAM subpopulations have a high overlap in cell markers and only relatively recently immunohistochemical procedures [16,17] or transgenic mouse models [18,19] were developed to address their individual roles. We have shown that combining single cell transcriptomics, transgenic models and immunohistological techniques provides an unprecedented and therapeutically relevant insight into the heterogeneity of TAM and other myeloid-marker positive cells (termed TAMEP) [20].

In primary GBM, TAM density positively correlates with malignancy [21,22,23] and it was shown that TAM promoted tumor expansion by increasing GBM cell invasion [13,14,24]. However, the role of TAM in tumor recurrence is currently less well defined. Consequently, the contribution of individual TAM subsets, like resident microglia or MDM, to disease progression remains unclear. It was suggested that surgical debulking damages the blood–brain barrier and induces an inflammatory reaction resulting in altered cellular composition of the TME [25]. Consequently, more macrophages are recruited to the resected site [26]. In addition, standard radiotherapy or chemotherapy were discussed to alter the fraction of MDM in all TAM following the treatment of primary GBM [27].

To investigate the biology of tumor recurrence we developed a minimally invasive tumor relapse model by genetically engineering an established murine GBM cell line with the HSVTK suicide gene (mGBM-TK cells); the cell death mechanism is activated after ganciclovir (GCV) administration [28,29]. This model allows initial tumor growth and partial remission in deep areas of the brain, which is an advantage over the (few) existing recurrent GBM models, which, e.g., relied on superficial tumor cell application and subsequent surgical removal [30,31,32]. Furthermore, GCV dosing gives a handle to control the extent and the timing of GBM debulking. Notably, this model recapitulated the enhanced tumor cell invasiveness observed in human GBM at post treatment recurrence [33,34]. Application of this novel GBM relapse model in different mouse reporter strains for microglia or tumor-invading macrophages [18,20] showed an increase in the MDM population (as compared to microglial cells) upon tumor recurrence. This shift from microglia towards MDM was not affected by surgical intervention and was confirmed in a matched, patient-derived GBM model for primary and recurrent GBM with verified histological markers for myeloid cells.

## 2. Materials and Methods

### 2.1. Cell Culture and Ganciclovir Treatment In Vitro

Murine mGBM-TK cells were obtained by transduction of GL261 cells (NCI-Frederick) with lentiviral vectors encoding a recombinant HSVTK fused with GFP [35] and were cultured in DMEM containing 10% fetal bovine serum (catalog no. 102270-106, Thermo Fisher Scientific, Waltham, MA, USA), 1× MEM non-essential amino acids (catalog no. 11140-035, Thermo Fisher Scientific, Waltham, MA, USA), and 1% penicillin-streptomycin (catalog no. 151140-122, Thermo Fisher Scientific, Waltham, MA, USA). For functional testing with ganciclovir (GCV; catalog no. PHR1593, Sigma-Aldrich, Taufkirchen, Germany), the GBM-TK cells were seeded in a 24-well plate (5000 cells per well). After 24 h, cells were treated with 0.5 mg/mL GCV in the GCV-treated group or with PBS solution alone in the control group. Cell death was monitored throughout the following days. GBM stem cell (GSC) cultures GBM 20 and GBM29 were derived from biopsies of the primary or the recurrent GBM of the same human patient [36] and cultured in DMEM-F12 (catalog no. 11320-074, Thermo Fisher Scientific, Waltham, MA, USA) supplemented with 1× B27 (catalog no. 17504-044, Thermo Fisher Scientific, Waltham, MA, USA), 1% penicillin-streptomycin, 10 ng/mL epidermal growth factor (EGF, catalog no. 236-EG; Biotechne; Minneapolis, MN, USA), and 10 ng/mL fibroblast growth factor (FGF, catalog no. 100-18B PeproTech, Hamburg, Germany). All cells were maintained at 37 °C in a humidified atmosphere of 95% O_2_ and 5% CO_2_. GSCs were validated repeatedly by short tandem repeat (STR) fingerprinting (Eurofins Medignomix Forensik, Munich, Germany) and regularly tested for mycoplasma contamination by PCR.

### 2.2. Animals

All animal experiments were performed in compliance with the German National Guidelines for Animal Protection and conducted with the approval of the local animal care committee of the Government of Oberbayern (Regierung von Oberbayern; Az.55.2-1-54-2532). Microglia reporter mice *Cx3cr1*::creER2, R26-RFP were created by cross breeding Cx3cr1-cre/ERT2Litt/WganJ (RRID:IMSR_JAX: 021160), expressing a fusion protein of cre-recombinase and a modified estrogen-receptor (cre-ER2) under the *CX3CR1* promoter with a cre-reporter line (B6.Cg-Gt(ROSA)26Sortm9(CAG-tdTomato)Hze/J [37]). To label tumor-invading macrophages in the brain, the myeloid cell reporter Ccr2-eGFP/Cln/J (RRID:IMSR_JAX: 027619) was used. All mice were purchased from the Jackson Laboratory, bred on C57Bl/6J background and genotyping was performed as previously described. Immunodeficient Foxn1nu/nu or B6.129S6-Rag2tm1Fwa mice were used for xenografting of human GBM cells as described previously [38]. Animals were kept in suitable cages with ad libitum access to water and food in a 12 h light/dark cycle at the standardized animal house of the Walter Brendel Centre for Experimental Medicine, LMU Munich. Mice were sacrificed at defined presymptomatic time points or at defined humane endpoints.

### 2.3. Tumor Implantation, Tamoxifen and GCV Treatment In Vivo

Mice received i.p. 7 µL/g body weight of a mixture of 0.1% xylazine (Rompun 2%; Bayer, Leverkusen, Germany) and 1.5% ketamine (Ketavet; Zoetis, Berlin, Germany) in 0.9% NaCl. A middle incision was made on the skin with a scalpel after disinfection with a 10% povidone iodine solution. To prevent the animals’ corneas from drying out, their eyes were covered with Bepanthen cream. Mice were immobilized on a stereotactic frame in a flat-skull position. After drilling a hole into the skull with a 23 G needle tip (coordinates 1.0 m anterior and 1.5 mm right of the bregma), 1 μL of cells (1 × 10^5^ murine GBM cells/μL or 5 × 10^4^ human GBM cells/μL in a supplement-free medium) was slowly injected within two minutes with a 22 G Hamilton syringe at a depth of 3 mm (the syringe was vertically inserted 4 mm and retracted 1 mm). Finally, the syringe was retracted 1 mm/min, and the skin was carefully sutured. For microglia tracing, *Cx3cr1*::creER2, R26-RFP mice received i.p. 75 mg/kg/d tamoxifen (dissolved in corn oil) in an intraperitoneal injection. The tamoxifen injection was performed three consecutive days. For in vivo depletion of GBM-TK cells, GCV was applied i.p. on four consecutive days from 14 to 17 DPO at 50 mg/kg/d.

### 2.4. Mouse Brain Tissue Preparation

Mice were transcardially perfused with 1× PBS (Pharmacy, University Clinics, LMU Munich) followed be 4% PFA solution (Sigma Aldrich, St. Louis, MO, USA) under anesthesia. The brain was carefully removed and incubated with 4% PFA at 4 °C for 24 h and then immersed in 30% sucrose until the brain sank to the bottom of the tube. The brain was then embedded in Cryomatrix (Cat. 6769006; Thermo Fisher Scientific, Waltham, MA, USA) and frozen with 2,2,4-Trimethylpentane in liquid nitrogen. Sequential and horizontal 40 μm-thick sections were prepared using a horizontal sliding microtome. Floating sections were stored in 24-well plates filled with cryoprotectant (ethylene glycol, glycerol, and 1× PBS pH 7.4 with a ratio 1:1; two at) at −20 °C and protected from light.

### 2.5. H&E Staining, Tumor Size Quantification and Scoring of Invasiveness

Hematoxylin and Eosin (H&E)-staining was performed and tumor size determination was done as previously described [20]. In short, brains were sectioned and every 12th axial section at 1.8 to 4.2 mm from the dural surface was sampled (representing the area that was infiltrated by the tumor). Tumor volume was quantified according to the Cavalieri principle by determining the tumor area using the Axiovision Rel. 4.9 software (Carl Zeiss, Jena, Germany) in every sampled brain slice. Stereotactical coordinates of brain slices containing GBM were determined and used to calculate a Z-axis of the experimental brain tumor. This Z-axis was multiplied with the average brain tumor area per brain-section to obtain a tumor volume per animal. Invasive scores were assessed on H&E stained sections as previously described [39]. For every mouse, every 6th axial brain section containing tumor was given an invasive score from 0 to 3 as follows: a score of 0 means no histological cell invasion from the tumor mass is observed; a score of 1 represents a more extensive, connected group of invading GBM cells; a score of 2 describes smaller scattered groups of invading GBM cells; and a score of 3 indicates single, scattered, highly invasive GBM cells.

### 2.6. Immunofluorescence Staining and Quantification on Mouse Brain Sections

Floating sections were washed three times for five minutes in PBT (0.1% Tween-20 in 1× PBS) and then incubated in blocking buffer (5% normal donkey serum and 0.3% Triton X-100 in 1× PBS) for one hour at room temperature. Samples were incubated with the following primary antibodies: goat anti-GFP (1:400; catalog no. R1091P; OriGene Technologies, Rockville, MD, USA), rat anti-CD49d (1:50; catalog no. 103701; BioLegend, San Diego, CA, USA), goat anti-Iba1 (1:400; catalog no. ab5076; Abcam, Cambridge, UK) and rabbit anti-TMEM119 (1:100; catalog no. ab209604; Abcam) overnight at 4 °C. The next day, the sections were incubated for 2 h at room temperature with the following secondary antibodies: biotin-labeled donkey anti-rat or anti-rabbit (1:250; catalog no. 712-065-150; 711-065-152; Jackson Immuno-Research, West Grove, PA, USA) and/or for 1 h at room temperature with streptavidin-AF 647 or –AF488 (1:500; catalog no. 016-600-084; 016-540-084; Jackson Immuno-Research). Alternatively, sections were directly incubated for 2 h at room temperature with the secondary antibodies: donkey anti-goat AF488 or Cy3 (1:500; catalog no. 711-545-152; 705-165-147; Jackson Immuno-Research) and donkey anti-rat 647 (1:500; 712-605-153; Jackson Immuno-Research). All antibodies were diluted in blocking buffer. Nuclei were stained with 4’,6-Diamidino-2-phenylindol (DAPI; 1:10,000; Sigma Aldrich) for two minutes and washed three times in PBT. Finally, sections were mounted in a fluorescent mounting medium (catalog no. S3023; Dako, Agilent Technologies, Santa Clar, CA, USA).

For the quantification of the macrophage or microglia subpopulation in all tumor-associated myeloid cells (TAM), immunostainings were photographed on a Leica TCS SP8 confocal microscope at 40× magnification. Four images per tumor area on three random brain tumor sections per animal were obtained resulting in 12 images per animal. TAM were identified by Iba1-positivity, the microglia-subpopulation was identified by additional co-staining with the verified marker for tumor-associated microglia TMEM119 or by microglia-tracing in the *Cx3cr1*::creER2, R26-RFP mouse strain. The MDM subpopulation was identified by additional co-labeling with the verified macrophage marker CD49d or by tracing tumor-invading macrophages in the Ccr2-eGFP mouse. Numbers of single marker- or marker double-positive cells (validated for DAPI-positive nuclei) were counted by the multi-point tool in ImageJ. Finally, the percentages of macrophage or microglia subpopulations shown in the respective graphs were obtained by normalization of the respective cell numbers to total numbers of Iba1-positive TAM.

### 2.7. Statistical Analysis

All statistical analyses were performed using the GraphPad Prism 7 software (GraphPad Software, San Diego, CA, USA). The number of individuals, replicates, and or repetition of independent experiments are indicated in the figure text. An unpaired Student’s *t*-test was used when two independent groups were compared. One-way ANOVA together with a Newman–Keuls post hoc test was used in graphs comparing more than two groups. The Log-rank (Mantel–Cox) test was used to determine statistical significance in the survival experiment. The criterion for statistically significant differences was *p* < 0.05. *p*-values as shown in figures are: *, *p* < 0.05; **, *p* < 0.01; ***, *p* < 0.001; ****, *p* < 0.0001; and NS, not significant.

## 3. Results

### 3.1. Establishing a Non-Invasive Mouse Model for GBM Recurrence

**Figure 1:**
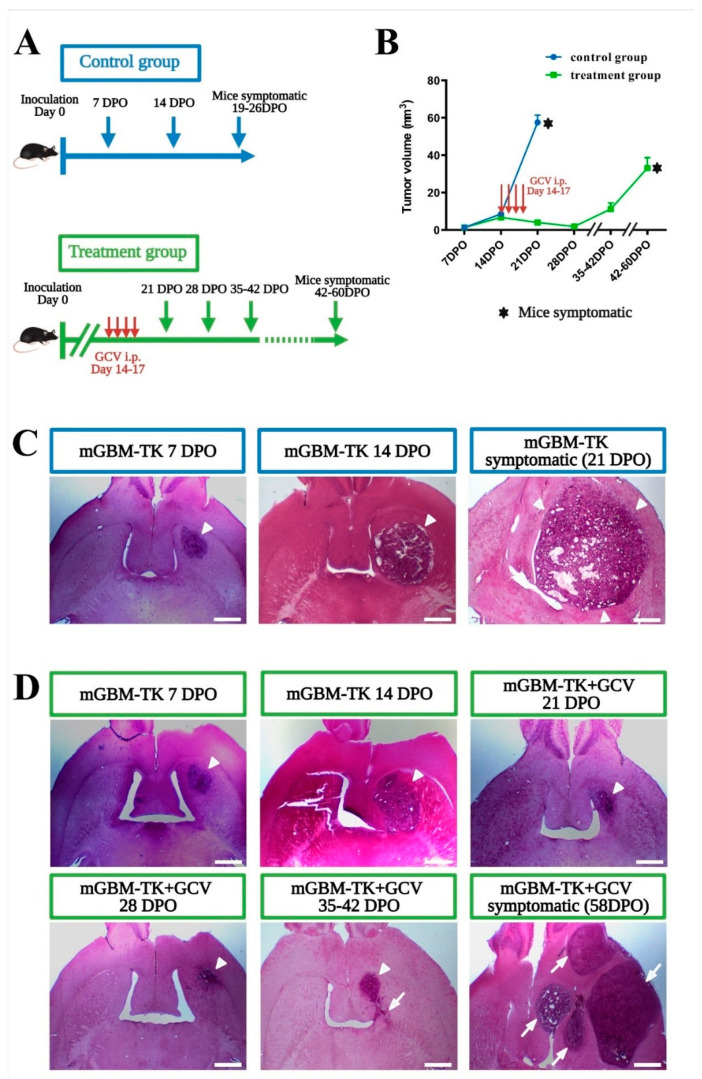
Establishing a minimally invasive mouse model for GBM recurrence. (**A**) Experimental schedules for the orthotopic implantation of the murine GBM cell line with the HSVTK suicide gene (mGBM-TK cells) and in vivo gangciclovir (GCV) treatment. In the treatment group, GCV was applied i.p. from 14 to 17 days post operation (DPO) daily while the control group remained untreated. Tumor volumes were assessed at 7, 14, 21, 28, 35–42 DPO and at the respective humane endpoints. (**B**) Tumor volumes of mice from control- and treatment-group are compared over time producing a growth curve. In the control group, tumor size increased by steady exponential growth while in the treatment group, tumor size increased initially, but decreased after GCV treatment until 28 DPO. Subsequently, tumor volume increased again exponentially until mice reached the pre-defined experimental endpoint (became symptomatic). (**C**,**D**) Representative H&E-stained sections illustrate tumor size and morphology obtained at the different time points in the untreated control group (**C**) or the GCV treatment group (**D**). Arrowheads indicate the tumor mass growing at the site of implantation. Arrows indicate mGBM-TK cells in additional areas at recurrence leading to a multifocal appearance at humane endpoints. Numbers of mice in the untreated control group were *n* = 7 at 7 DPO, *n* = 9 at 14 DPO and *n* = 10 at 21–26 DPO (humane endpoint) and for the GCV treatment group *n* = 6 at 7 DPO, *n* = 6 at 14 DPO, *n* = 10 at 21 DPO, *n* = 4 at 28 DPO, *n* = 6 at 35–42 DPO and *n* = 7 at humane endpoint. Values in the graph (**B**) are represented as the mean ± SEM. Scale bars represent 1 mm (**C**,**D**).

The mGBM-TK cell line efficiently underwent cell death after in vitro GCV application (Figure S1A,B). After implantation of the mGBM-TK cells into the striatum of syngeneic wildtype mice (C57BL/6J), intraperitoneal (i.p.) GCV treatment from 14 to 17 days post operation (DPO) resulted in a very strong reduction of the tumor mass at 21 DPO (Figure S1C–E). Next, we performed a survival study using mGBM-TK cells implanted mice and compared GCV treatment (14 to 17 DPO) with controls (without GCV). Average survival of GCV-treated mGBM-TK mice was significantly prolonged to 55 DPO as compared to 21 DPO in the untreated controls (Figure S1E). In order to compare the pathological history in both groups, we next sacrificed mice every 7 days over the course of the disease and analyzed the brain tumors generated by mGBM-TK cells by histology (Figure 1A). In the untreated control group, the tumor mass gradually increased from 7 over 14 until 19–26 DPO, when mice became symptomatic (Figure 1B,C). In the recurrent mGBM derived from mGBM-TK cells implanted and treated with GCV (at 14 to 17 DPO) the tumor volume constantly decreased from 14 over 21 to 28 DPO (Figure 1B) and showed the histopathological pattern of a largely compact tumor mass (Figure 1D; arrowhead). After that, the remaining mGBM-TK cells began to grow again from clusters of surviving cells (Figure 1D, arrows) until 42–60 DPO when mice became symptomatic (Figure 1B,D and Figure S1D). The total volume of recurrent tumors at the humane endpoints was smaller (Figure 1B) than in the primary tumors (untreated controls). Interestingly, the recurrent mGBM had a multifocal histopathological appearance (Figure 1D; arrows) whereas primary mGBM controls usually presented as a single tumor mass (Figure 1C; arrowheads). In summary, our pharmacological mGBM-TK cell implantation model led to tumor relapse after near complete remission, which recapitulates GBM recurrence after surgical debulking.

### 3.2. Increased Invasiveness in the Recurrent GBM Mouse Model

**Figure 2:**
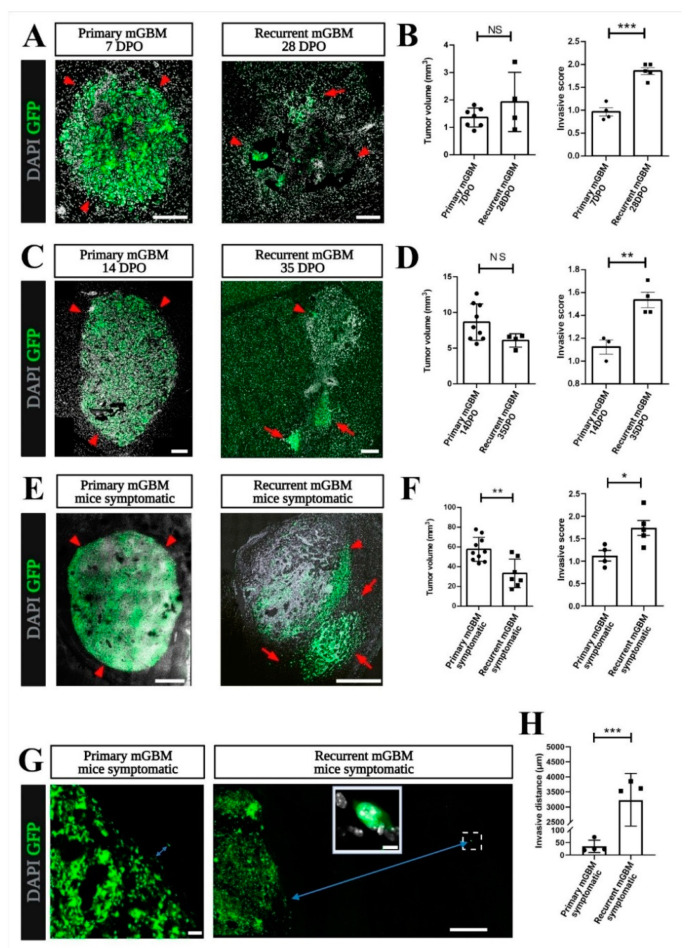
GBM cells in the recurrent GBM model show increased invasiveness. (**A**–**H**) Immunostainings against green fluorescent protein (GFP) on tissue sections of the primary mGBM and the recurrent mGBM model was performed to localize the invasive GFP-positive tumor cells in combination with nuclear DAPI staining to indicate cell dense tumor mass. Representative images of stained tumor sections are shown for primary and recurrent mGBM at (**A**) early (**C**) intermediate and (**E**) late stage of tumor development. (**B**,**D**,**F**) Comparisons of the H&E tumor volumes by the Cavalieri method showed no significant differences when comparing tumor sizes at (**B**) early and (**D**) intermediate stage but tumor volumes were different at symptomatic (**F**) end stage of tumor growth. Number of mice analyzed for tumor volumes were in (**B**) *n* = 7 for primary mGBM at 7 DPO and *n* = 4 for recurrent mGBM at 28 DPO in (**D**) for primary GBM *n* = 9 at 14 DPO and *n* = 4 for recurrent mGBM at 35 DPO and in (**F**) *n* = 10 for primary mGBM and *n* = 7 for recurrent mGBM. Interestingly, quantification of the invasive scores on GFP-immunostained cells in (**B**) early, (**D**) intermediate and (**F**) late stage of recurrent mGBM was significantly higher compared to the respective primary mGBM group. Number of mice analyzed for the invasive scores were in (**B**) *n* = 4 for primary mGBM at 7 DPO and *n* = 5 for recurrent mGBM at 28 DPO, in (**D**) *n* = 3 for primary mGBM at 14 DPO and *n* = 4 for recurrent mGBM at 35 DPO and in (**F**) *n* = 10 for primary mGBM and *n* = 7 for recurrent mGBM. (**G**) Representative images show GFP-positive, invasive mGBM-TK cells in end stage tumors of primary or recurrent mGBM. The blue arrows indicate the invasive distance of a single cell from the tumor border. The magnified inset is a confocal maximum projection of a single invasive tumor cell in the recurrent mGBM (dashed rectangle). (**H**) Invasive distances (from the tumor border to the invasive cells) were measured at the pre-defined experimental endpoint turned out to be significantly higher (by two orders of magnitude) in recurrent (*n* = 3) compared to primary mGBM (*n* = 4). Statistical significance was calculated according to *t*-test, * *p* < 0.05, ** *p* < 0.01, *** *p* < 0.0005. Each dot in the diagrams represents the average statistical value obtained from one mouse. Scale bars represent 200 µm (**A**,**C**), 800 µm (**E**), 50 µm ((**G**), primary mGBM), 10 µm ((**G**), inserted image) and 500 µm ((**G**), recurrent mGBM).

In patients, GBM recurrence is often seen in brain regions that are relatively close to the site of the primary tumor but can also occur at distant intracerebral locations [40]. Likewise, in our relapse model, tumors recurred both at the original glioma site and at additional sites in a multifocal appearance. This indicated that mGBM-TK cells, throughout the process of tumor relapse, became more invasive than the primary tumor cells [39]. For comparison of the invasion phenotype, we analyzed tumor volumes from primary and recurrent at early, mid, and late stage of in vivo tumor growth. The earliest time point of tumor relapse we defined at 28 DPO when tumor volume after GCV treatment was smallest (Figure 1B). Next, we defined the endpoint of tumor development when mice became symptomatic in primary (19–26 DPO) or in the recurrent mGBM model (42–60 DPO). In addition, an intermediate time point was chosen for comparison of tumors at exponential growth, that was at 14 DPO or at 35 DPO for primary or recurrent mGBM, respectively (Figure 1B). By histopathological inspection at all the three stages of tumor growth the individual tumor volumes were assessed and the invasive phenotypes were scored [39]. Quantification of the Cavalieri tumor volumes confirmed that in the early stage of tumor growth, there was no significant difference in tumor size between the primary (7 DPO) and the recurrent mGBM (28 DPO) showing 1.70 and 1.42 mm^3^, respectively (Figure 2A,B). However, the invasive score of the recurrent mGBM was significantly higher (Figure 2B) than the invasive score of the primary mGBM (1.86 compared to 0.97) showing scattered groups of mGBM-TK cells that separated from the original tumor mass (Figure 2B; arrows). In the intermediate stage at exponential tumor growth (Figure 2C,D), tumor sizes in primary mGBM (14 DPO) and in recurrent mGBM (35 DPO) were again similar (6.45 or 7.95 mm^3^). The invasive score of the recurrent mGBM (35 DPO; invasive score: 1.54) was, however, significantly higher (Figure 2D) than in the primary mGBM (14 DPO; invasive score: 1.12) model. At late stage of tumor growth (when mice became symptomatic and experiments were terminated; Figure 2E), the tumor sizes of the recurrent mGBM were profoundly smaller than in the primary mGBM model (27.47 compared to 54.47 mm^3^). Notably, the invasive score of the recurrent mGBM (1.74) at this stage was strongly elevated as compared to the primary mGBM (invasive score: 1.12; Figure 2F).

We also compared the maximum invasive distances of single (GFP-positive) tumor cells in primary and recurrent mGBM (defined as trajectories between the border of the compact tumor mass located at the stereotactic coordinates of the implantation site and the most distantly invading cells). Strikingly, tumor cells of the recurrent mGBM model infiltrated very far into the brain (Figure 2G,H), while this was never observed for primary mGBM. Consequently, the average invasive distance in recurrent mGBM (Figure 2E) was much larger than in primary mGBM (3225 µm compared to 34.75 µm). This is underlined by the finding that only in the recurrent mGBM model distant relapse (like, e.g., in the cerebellum) was repetitively observed (and unequivocally confirmed by GFP-positivity; Figure S2A,B).In summary, we established a novel, minimally invasive mouse model for recurrent GBM, which recapitulates the pattern of increased invasiveness (even to distant sites) and the multifocal pattern of relapsing tumors that can be observed in patients [41,42,43].

### 3.3. Monocyte-Derived Macrophages Make Up the Majority of TAM in Recurrent GBM

**Figure 3:**
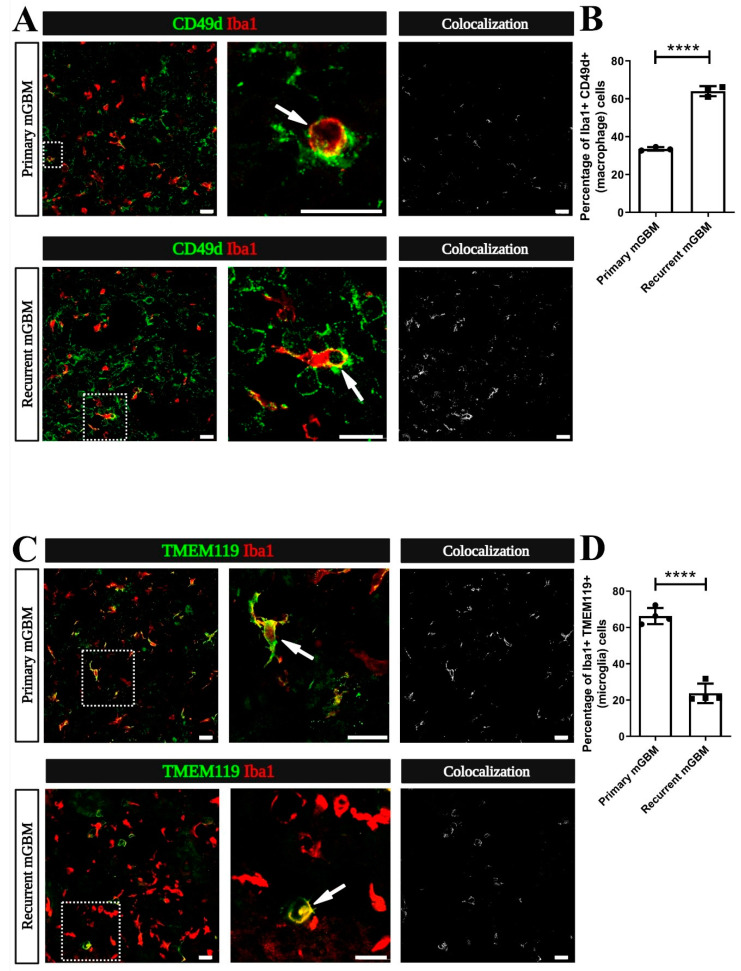
The ratio of monocyte-derived macrophages (MDM) to resident microglia is increased in recurrent GBM. (**A**–**D**) Sections from GBM models reaching the pre-defined experimental endpoint (symptomatic mice) were co-immunostained for the myeloid cell marker Ionized Calcium-Binding Adapter Molecule 1 (Iba1; red), the MDM-marker CD49d or for the microglia-marker Transmembrane Protein 119 (TMEM119) Staining for CD49d or TMEM119 is presented in green. (**A**) Representative micrographs of TAM in primary and recurrent murine GBM (mGBM) are shown in the left panel. A CD49d^+^Iba1^+^ double-positive (yellow) MDM (dashed rectangle) is magnified in the middle panel (arrow). In the right panel, Colocalization figures, obtained by the RG2B Colocalization plugin from ImageJ, indicate an increase of the CD49d^+^Iba1^+^ macrophage staining in recurrent compared to primary mGBM. (**B**) Quantification of the numbers of CD49d^+^Iba1^+^ macrophages was performed and compared to the total number of Iba1-positive TAM. The percentage of MDM in all TAM increased from 33.54% in primary mGBM to 64.01% in recurrent mGBM. Number of mGBMs analyzed was *n* = 3 per group. (**C**) Representative images depict tumor-associated TMEM119+Iba1+ double-positive (yellow) microglia (dashed rectangle) in primary and recurrent GBM (left panel). A magnified colabeled microglia is shown in the middle panel (arrows). In the right panel, “Colocalization” figures indicate a decrease of the TMEM119^+^Iba1^+^ microglia staining in recurrent compared to primary mGBM; (**D**) Quantification of the number of TMEM119^+^Iba1^+^ microglia was performed compared to the total number of Iba1-positive TAM. The percentage of microglia in all TAM decreased from 66.27% in primary GBM to 23.74% in recurrent mGBM. Number of mGBMs was *n* = 4 per group. (**B**,**D**) Statistical significance was calculated by student’s *t*-test, **** indicating *p* < 0.0001. Each dot in the diagrams represents the average statistical value obtained from one mouse. Scale bars represent 20 µm (**A**,**C**).

TAM (comprising CNS-resident microglia and monocyte-derived macrophages) can promote tumor cell invasion [13,15,24]. Therefore, we investigated if the increased invasiveness in our recurrent GBM model is associated with changes in the number or composition of TAM. Hence, we immunostained samples from the primary or recurrent mGBM models for markers identifying MDM (integrin subunit alpha 4; ITGA4 also designated as CD49d) and for Ionized Calcium-Binding Adapter Molecule 1 (Iba1), which indicates all TAM (both MDM and microglia) [17] (Figure 3A). Interestingly, we found that in recurrent mGBM Iba1-positive cells co-labeling for CD49d were much more abundant than in primary mGBM (Figure 3B). Notably, overall numbers of Iba1-positive cells were not significantly changed. Next, we used the established microglia marker Transmembrane Protein 119 (TMEM119) [16] to identify tumor-associated microglia in our mGBM models (Figure 3C). In correspondence with our data from CD49d immunolabeling we found that Iba1 and TMEM119 co-labeled cells were much less abundant in recurrent as compared to primary mGBM (Figure 3D). Overall, we found that tumor-invading macrophages account for the majority of TAM in models for recurrent GBM whereas primary GBM largely harbor microglia.

We used different strategies to control for the specificity of our immunolabeling procedures. In a first series of control experiments, we used a *Cx3cr1*::creER2, R26-RFP mouse strain together with an established tamoxifen pulse-chase protocol [19] to exclusively label microglia in brain specimen (Figure S3A). Here, orthotopic implantation of GBM-cells was performed 28 days after tamoxifen treatment (this long chase period assures that, in the GBM microenvironment, RFP is expressed by microglia but not MDM) [19]. Tumors were allowed to grow for 7 days, and tissue was analyzed by immunostaining for CD49d or TMEM119 (Figure S3B). As expected CD49d staining was mutually exclusive with RFP-expression in Iba1-positive cells (Figure S3C). TMEM119 and the RFP tracer overlapped very reliably in experimental tumors (Figure S3B) and these TMEM119^+^RFP^+^ microglial cells accounted for approximately 25% of all Iba1-positive cells of the tumor parenchyma (Figure S3C). Similar data were obtained with the alternative immunolabeling paradigm (Figure S3C; 25% of all Iba1^+^ TAM were CD49d^−^ RFP^+^ microglia). Next, we used the CCR2-GFP mouse strain [18] to re-evaluate the specificity of our MDM marker CD49d and the microglial marker TMEM119 with another, independent transgenic model (Figure S3D). This fully confirmed MDM as CD49d-positive and TMEM119-negative cells and corroborated that 25% of all TAM (at seven days of glioma-expansion) are contributed by microglial cells (Figure S3D–F). Altogether, these different controls verified CD49d and TMEM119 as specific makers for MDM or microglia in the GBM microenvironment and thereby confirmed that models for primary or relapsing GBM differ in phagocyte composition.

### 3.4. Signals from GBM Relapse and Not Surgical Intervention Itself Attract Peripheral Macrophages

It was suggested that surgical resection might alter the immune cell composition of relapsing GBM [26,44,45]. A transient influx of MDM into the resection cavity appears conceivable, as removal of the tumor mass inevitably damages the surrounding tissue. We used our model for pharmacological debulking of gliomas to investigate if surgery has a lasting effect on the intracerebral accumulation of MDM. Therefore, we used the transgenic *Cx3cr1*::creER2, R26-RFP microglia-tracing model (applying the tamoxifen pulse-chase paradigm for the identification of microglia, as outlined above) [19,46] and implanted mGBM-TK cells.

**Figure 4:**
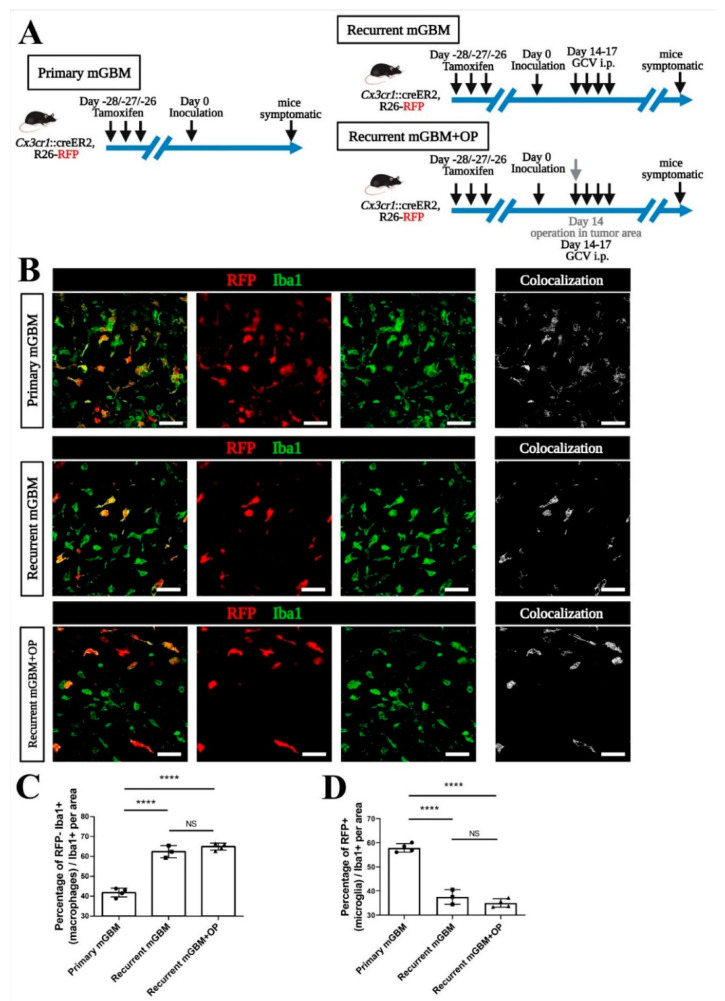
Signals from tumor cell relapse rather than surgical intervention attracts more macrophages into recurrent GBM. (**A**) Scheme for transgenic tracing of microglia in mouse GBM models. The *Cx3cr1*::creER, R26-RFP mouse model with a tamoxifen pulse-chase protocol was used for tracing of microglia. Tamoxifen was given at days 28, 27 and 26 before implantation of the murine GBM cell line carrying the HSVTK suicide gene (mGBM-TK). In recurrent murine GBM (mGBM), GCV was given at day 14, 15, 16 and 17 days post operation (DPO). All mice were sacrificed at the pre-defined experimental endpoint (when animals became symptomatic). In the recurrent mGBM + OP (operation) group, surgical debulking of the GBM tumor mass was modeled by performing an aspiration biopsy. (**B**) Mouse GBMs were sectioned and immunostained. Representative micrographs for co-immunostainings against Ionized Calcium-Binding Adapter Molecule 1 (Iba1) and red fluorescent protein (RFP)-traced microglia in “primary mGBM”, “recurrent mGBM” or “recurrent mGBM + OP” are shown. All TAM are stained for Iba1 in green, RFP-traced microglia are presented in red and co-labeled microglia in yellow. Colocalization figures on the right for RFP^+^Iba1^+^ microglia staining indicate a decrease in recurrent mGBM compared to primary mGBM. (**C**,**D**) Quantification of the number of RFP^−^Iba1^+^ cells indicating macrophages (**C**) or RFP^+^Iba1^+^ cells indicating microglia (**D**) was performed and compared to total numbers of all Iba1-positive TAM. We found an increase in the macrophage population and a decrease in the microglia population in all TAM of “recurrent mGBM” and “recurrent GBM + OP” compared to “primary mGBM”. Number of animals was *n* = 4 for primary GBM, *n* = 3 for recurrent GBM and *n* = 4 for recurrent GBM +OP. Statistical significance was calculated by one-way ANOVA with post hoc test (**C**,**D**), **** refers to *p* < 0.0001, NS refers to no significance. Each dot in the diagrams represents the average statistical value obtained from one mouse. Scale bars are 20 µm (**B**).

Again, primary or recurrent mGBM were generated from the same mGBM-TK cell implantation with and without i.p injection of GCV (Figure 4A). In a third experimental group (referred to as recurrent mGBM + OP), a surgical intervention (taking a large biopsy of the tumor mass) was performed together with the GCV treatment. Confocal analysis of immunofluorescence and transgenic markers (Figure 4B) revealed that the majority of Iba1-positive TAM in primary GBM were RFP-positive microglia (a facilitated view on all colocalizing pixels for the confocal channels recording Iba1 and RFP is given in the micrographs termed “Colocalization”; Figure 4B). In contrast, the majority of TAM observed in recurrent mGBM were RFP^−^Iba1^+^ MDM (Figure 4B–D). Strikingly, the neurosurgical intervention (in the experimental group recurrent mGBM + OP) did not have an enduring effect on the TAM-composition in relapsing GBM, which remained similar to the (entirely non-invasive) pharmacological debulking by GCV alone (Figure 4B–D). In summary these findings suggest that signals released specifically by the GBM relapse are sufficient to alter the macrophages-to-microglia ratio in recurrent GBM while surgery does not mediate an additional, durable effect on TAM composition.

### 3.5. Increased Macrophage Attraction Is Preserved in a Patient-Derived Xenograft Model for Recurrent GBM

**Figure 5:**
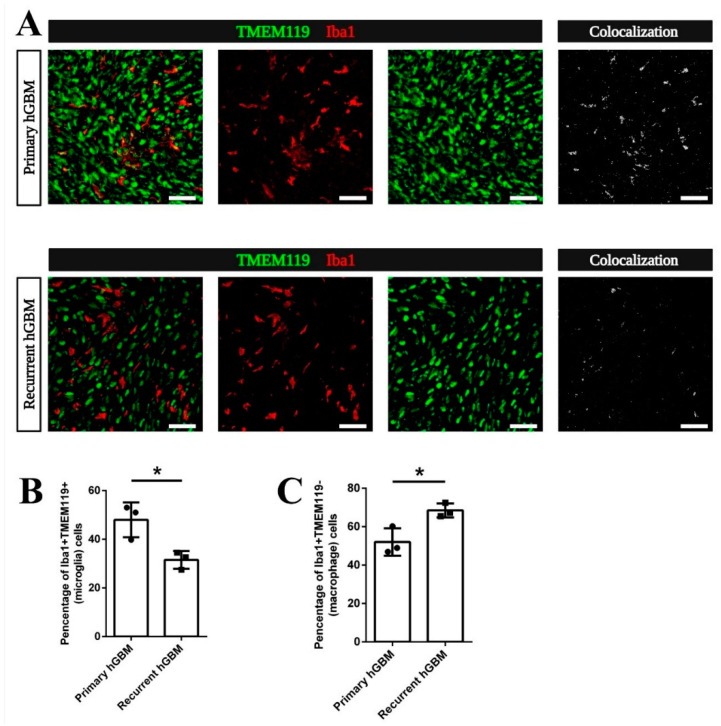
The increased macrophage-to-microglia ratio in recurrent GBM is hardwired to the relapsing tumor cell. (**A**) Mouse GBM tissue obtained by orthotopic implantation of primary or recurrent human GBM (hGBM) cells of the same patient was co-immunostained for Transmembrane Protein 119 (TMEM119) and Ionized Calcium-Binding Adapter Molecule 1 (Iba1). Double fluorescent pictures are shown on the left, red TMEM119 staining in the middle and green Iba1-stained TAM in green are shown in the right panel. Co-stained tumor-associated microglia display in yellow on the left. For better visualization, the “Colocalization” figures for TMEM119^+^Iba1^+^ costainings indicate that tumor-associated microglia are decreased. (**B**) Quantification of the number of TMEM119^+^Iba1^+^ cells was performed and compared to total number of Iba1-positive myeloid cells. This showed a significant decrease in the percentage of microglia in all TAM of recurrent hGBM compared to primary hGBM. (**C**) In contrast, the percentage of TMEM119^−^Iba1^+^ cells indicating MDM in all TAM is increased in recurrent hGBM compared to primary hGBM. Number of animals was *n* = 3 for both groups. Statistical significance according to *t*-test (**B**,**C**), * refers to *p* < 0.05. Each dot in the diagrams represents the average statistical value obtained from one mouse. Scale bars are 20 µm (**A**).

To investigate if the relative increase of MDM among TAM in recurrent GBM is caused by signals released from dying cells in the recurrent mGBM relapse model or is initiated by MDM chemoattractants preponderantly released from live recurrent GBM cells, we orthotopically implanted GBM stem-like cells (GSC) derived from a primary (GBM20) or a recurrent GBM (GBM29) of the same patient [36,38,47]. Both, GBM20 and GBM29 were left to expand until mice became symptomatic and sections were co-stained for Iba1 and TMEM119 (Figure 5A). Interestingly, tumor-associated microglia (co-expressing Iba1 and TMEM119; as specifically visualized in the micrograph termed “Colocalization” in Figure 5A) contributed significantly more to the total number of TAM in xenografted primary hGBM than in recurrent hGBM (Figure 5B). Coherently, the fraction of Iba1^+^TMEM119^−^ GBM-invading macrophages among all TAM was significantly increased in recurrent hGBM versus primary hGBM (Figure 5C). Hence, this model recapitulated the switch from a higher density of microglial TAM in primary GBM towards an increased density of MDM in recurrent GBM without an intermittent phase of pharmacological debulking (resulting in massive GBM cell death). This indicates important differences in immune-signaling cues of viable GBM at initial tumor growth and recurrence.

## 4. Discussion

Our data suggest that the specific biological characteristics of primary or recurrent GBM, but not surgery or cell death signaling, determine the contribution of microglia or MDM to the tumor microenvironment. This conclusion was based on the results obtained with both pharmacological debulking and patient-derived GSCs using a matched pair of primary and recurrent tumor cells. Our finding is in line with recent studies using single-cell profiling of myeloid cells in patient GBM [45]. These authors show that microglia-derived TAM are predominant in newly diagnosed tumors but are outnumbered by MDM following tumor recurrence.

While this shift towards tumor-invading macrophages was also observed in tumor recurrence after radiotherapy [27] it could not be resolved if this was a cause or a consequence of standard therapy against primary GBM. The advantage of our pharmacological mGBM-TK implantation model is that we can investigate tumor regrowth from GBM cells escaping a tumor cell specific death paradigm without therapeutic interventions that would also involve the parenchyma.

We observed that recurrent GBM were more invasive than primary GBM and often generated multifocal tumors at distant sites. Invasion is a pathological hallmark of GBM and likely a main reason for therapy failure [48,49]. Comparison of transcriptomic profiles from primary and patient-matched recurrent GBM suggested an evolutionary mechanism promoting therapy resistance and tumor recurrence [50,51]. Limitations for the interpretation of such approaches, however, are small patient cohorts [52]. Furthermore, in patient-derived samples it is usually difficult to analyze the consecutive genetic mechanisms of mutation and selection that occur during recurrence [10,40]. Our recurrent GBM models will allow dissecting the different microevolutionary steps promoting GBM relapse after pharmacological debulking alone or in conjunction with established therapeutic treatments.

Previous studies addressing tumor recurrence in preclinical models, e.g., used surgical removal of superficially implanted tumor into the cortex, which is not a common location for human GBM [26,31,32]. Moreover, the invasive nature of the surgical intervention causes damage that is highly variable from experiment to experiment. In other studies, whole brain irradiation was used to investigate tumor relapse with a dose that allowed the tumor to regress [53]. However, this method is largely different from clinical standard treatment. Usually, radiotherapy is applied after maximal surgical resection of GBM and irradiation is strictly directed towards an identified tumor target area [54,55]. In this study, we utilized the HSVTK suicide gene to establish a novel minimally invasive recurrent GBM mouse model. This new model recapitulated the whole process of glioma recurrence and demonstrated heterogeneous pathological features. Pharmacological treatment of mice with GCV i.p. efficiently killed mGBM-TK cells without side effects on the tumor microenvironment. By implanting stably transduced mGBM-TK cells into the mouse brain, we set up a system to induce cell death in dividing cells after GCV treatment. By controlling the GCV dose and narrowing the treatment window, we made sure that sufficient non-dividing cells at the time of treatment survive to produce a tumor to relapse after variable periods. We had previously shown that normal brain cells transduced by HSVTK in situ are not killed by GCV prodrug treatment, excluding bystander effects [56,57], while tumor cells were efficiently reduced [35,58].

Orthotopic implantation is a versatile method allowing to induce tumor relapse at various, pathologically relevant sites in the brain [59]. The extent and time course of tumor reduction can be closely controlled by induced cell death through the GCV concentration applied. By this, our relapse model can be executed in a scalable manner controlling number of cells implanted and measuring exact tumor volume upon remission.

## 5. Conclusions

We established a new minimally invasive mouse model for GBM relapse. We found that recurrent tumors were more aggressively invasive than primary GBM. Additionally, we found that the recurring tumors had a higher number of MDM and a lower number of microglia in the entire population of TAM. This shift in immune-composition of GBM appeared to be independent from cell-death signaling or surgical intervention. This model provides the means to thoroughly dissect the entire process of tumor relapse under defined conditions and represents an efficient tool for preclinical studies aiming to improve the therapeutic management of relapsing GBM.

## Data Availability

Supporting information and data on primary GBM cell cultures can be found at ArrayExpress and at European Nucleotide Archive (E-MTAB-7649, E-MTAB-7649, E-MTAB-9341, E-MTAB-9343, ENA: PRJEB24104) and in Mastrella et al., 2019 [38], Kälin et al., 2021 [20] and Volmar et al., 2021 [47].

## Supplementary Materials

The following are available online at https://www.mdpi.com/article/10.3390/cancers13143636/s1, Figure S1: mGBM-TK cells are efficiently killed by GCV treatment leading to prolonged survival of orthotopically implanted mice, Figure S2: Tumor recurrence is found throughout the brain in local and distant sites, Figure S3: TMEM119 and CD49d specifically distinguish tumor-associated microglia from monocyte derived-macrophages in mouse GBM.

## References

[1] Louis D.N., Perry A., Reifenberger G., von Deimling A., Figarella-Branger D., Cavenee W.K., Ohgaki H., Wiestler O.D., Kleihues P., Ellison D.W. (2016). The 2016 World Health Organization Classification of Tumors of the Central Nervous System: A summary. Acta Neuropathol..

[2] Weller M., van den Bent M., Preusser M., Le Rhun E., Tonn J.C., Minniti G., Bendszus M., Balana C., Chinot O., Dirven L. (2021). EANO guidelines on the diagnosis and treatment of diffuse gliomas of adulthood. Nat. Rev. Clin. Oncol..

[3] Stupp R., Mason W.P., van den Bent M.J., Weller M., Fisher B., Taphoorn M.J., Belanger K., Brandes A.A., Marosi C., Bogdahn U. (2005). Radiotherapy plus concomitant and adjuvant temozolomide for glioblastoma. N. Engl. J. Med..

[4] Weller M., van den Bent M., Tonn J.C., Stupp R., Preusser M., Cohen-Jonathan-Moyal E., Henriksson R., Le Rhun E., Balana C., Chinot O. (2017). European Association for Neuro-Oncology (EANO) guideline on the diagnosis and treatment of adult astrocytic and oligodendroglial gliomas. Lancet Oncol..

[5] Bernstock J.D., Mooney J.H., Ilyas A., Chagoya G., Estevez-Ordonez D., Ibrahim A., Nakano I. (2019). Molecular and cellular intratumoral heterogeneity in primary glioblastoma: Clinical and translational implications. J. Neurosurg..

[6] Birzu C., French P., Caccese M., Cerretti G., Idbaih A., Zagonel V., Lombardi G. (2020). Recurrent Glioblastoma: From Molecular Landscape to New Treatment Perspectives. Cancers.

[7] Brennan C.W., Verhaak R.G., McKenna A., Campos B., Noushmehr H., Salama S.R., Zheng S., Chakravarty D., Sanborn J.Z., Berman S.H. (2013). The somatic genomic landscape of glioblastoma. Cell.

[8] Burger P.C., Dubois P.J., Schold S.C., Smith K.R., Odom G.L., Crafts D.C., Giangaspero F. (1983). Computerized tomographic and pathologic studies of the untreated, quiescent, and recurrent glioblastoma multiforme. J. Neurosurg..

[9] De Bonis P., Anile C., Pompucci A., Fiorentino A., Balducci M., Chiesa S., Lauriola L., Maira G., Mangiola A. (2013). The influence of surgery on recurrence pattern of glioblastoma. Clin. Neurol. Neurosurg..

[10] Van Nifterik K.A., Elkhuizen P.H., van Andel R.J., Stalpers L.J., Leenstra S., Lafleur M.V., Vandertop W.P., Slotman B.J., Hulsebos T.J., Sminia P. (2006). Genetic profiling of a distant second glioblastoma multiforme after radiotherapy: Recurrence or second primary tumor?. J. Neurosurg..

[11] Tully P.A., Gogos A.J., Love C., Liew D., Drummond K.J., Morokoff A.P. (2016). Reoperation for Recurrent Glioblastoma and Its Association With Survival Benefit. Neurosurgery.

[12] Aldape K., Brindle K.M., Chesler L., Chopra R., Gajjar A., Gilbert M.R., Gottardo N., Gutmann D.H., Hargrave D., Holland E.C. (2019). Challenges to curing primary brain tumours. Nat. Rev. Clin. Oncol..

[13] Glass R., Synowitz M. (2014). CNS macrophages and peripheral myeloid cells in brain tumours. Acta Neuropathol..

[14] Hambardzumyan D., Gutmann D.H., Kettenmann H. (2016). The role of microglia and macrophages in glioma maintenance and progression. Nat. Neurosci..

[15] Audia A., Conroy S., Glass R., Bhat K.P.L. (2017). The Impact of the Tumor Microenvironment on the Properties of Glioma Stem-Like Cells. Front. Oncol..

[16] Bennett M.L., Bennett F.C., Liddelow S.A., Ajami B., Zamanian J.L., Fernhoff N.B., Mulinyawe S.B., Bohlen C.J., Adil A., Tucker A. (2016). New tools for studying microglia in the mouse and human CNS. Proc. Natl. Acad. Sci. USA.

[17] Bowman R.L., Klemm F., Akkari L., Pyonteck S.M., Sevenich L., Quail D.F., Dhara S., Simpson K., Gardner E.E., Iacobuzio-Donahue C.A. (2016). Macrophage Ontogeny Underlies Differences in Tumor-Specific Education in Brain Malignancies. Cell Rep..

[18] Chen Z., Feng X., Herting C.J., Garcia V.A., Nie K., Pong W.W., Rasmussen R., Dwivedi B., Seby S., Wolf S.A. (2017). Cellular and Molecular Identity of Tumor-Associated Macrophages in Glioblastoma. Cancer Res..

[19] Wieghofer P., Knobeloch K.P., Prinz M. (2015). Genetic targeting of microglia. Glia.

[20] Kälin R.E., Cai L., Li Y., Zhao D., Zhang H., Cheng J., Zhang W., Wu Y., Eisenhut K., Janssen P. (2021). TAMEP are brain tumor parenchymal cells controlling neoplastic angiogenesis and progression. Cell Syst..

[21] Glass R., Synowitz M., Kronenberg G., Walzlein J.H., Markovic D.S., Wang L.P., Gast D., Kiwit J., Kempermann G., Kettenmann H. (2005). Glioblastoma-induced attraction of endogenous neural precursor cells is associated with improved survival. J. Neurosci..

[22] Markovic D.S., Glass R., Synowitz M., Rooijen N., Kettenmann H. (2005). Microglia stimulate the invasiveness of glioma cells by increasing the activity of metalloprotease-2. J. Neuropathol. Exp. Neurol..

[23] Watters J.J., Schartner J.M., Badie B. (2005). Microglia function in brain tumors. J. Neurosci. Res..

[24] Markovic D.S., Vinnakota K., Chirasani S., Synowitz M., Raguet H., Stock K., Sliwa M., Lehmann S., Kälin R., van Rooijen N. (2009). Gliomas induce and exploit microglial MT1-MMP expression for tumor expansion. Proc. Natl. Acad. Sci. USA.

[25] Xue J., Zhao Z., Zhang L., Xue L., Shen S., Wen Y., Wei Z., Wang L., Kong L., Sun H. (2017). Neutrophil-mediated anticancer drug delivery for suppression of postoperative malignant glioma recurrence. Nat. Nanotechnol..

[26] Zhu H., Leiss L., Yang N., Rygh C.B., Mitra S.S., Cheshier S.H., Weissman I.L., Huang B., Miletic H., Bjerkvig R. (2017). Surgical debulking promotes recruitment of macrophages and triggers glioblastoma phagocytosis in combination with CD47 blocking immunotherapy. Oncotarget.

[27] Akkari L., Bowman R.L., Tessier J., Klemm F., Handgraaf S.M., de Groot M., Quail D.F., Tillard L., Gadiot J., Huse J.T. (2020). Dynamic changes in glioma macrophage populations after radiotherapy reveal CSF-1R inhibition as a strategy to overcome resistance. Sci. Transl. Med..

[28] Beltinger C., Fulda S., Kammertoens T., Meyer E., Uckert W., Debatin K.M. (1999). Herpes simplex virus thymidine kinase/ganciclovir-induced apoptosis involves ligand-independent death receptor aggregation and activation of caspases. Proc. Natl. Acad. Sci. USA.

[29] Tomicic M.T., Thust R., Kaina B. (2002). Ganciclovir-induced apoptosis in HSV-1 thymidine kinase expressing cells: Critical role of DNA breaks, Bcl-2 decline and caspase-9 activation. Oncogene.

[30] Bello L., Giussani C., Carrabba G., Pluderi M., Lucini V., Pannacci M., Caronzolo D., Tomei G., Villani R., Scaglione F. (2002). Suppression of malignant glioma recurrence in a newly developed animal model by endogenous inhibitors. Clin. Cancer Res..

[31] Kauer T.M., Figueiredo J.L., Hingtgen S., Shah K. (2011). Encapsulated therapeutic stem cells implanted in the tumor resection cavity induce cell death in gliomas. Nat. Neurosci..

[32] Hingtgen S., Figueiredo J.L., Farrar C., Duebgen M., Martinez-Quintanilla J., Bhere D., Shah K. (2013). Real-time multi-modality imaging of glioblastoma tumor resection and recurrence. J. Neuro Oncol..

[33] Jones T.S., Holland E.C. (2012). Standard of care therapy for malignant glioma and its effect on tumor and stromal cells. Oncogene.

[34] Lu K.V., Bergers G. (2013). Mechanisms of evasive resistance to anti-VEGF therapy in glioblastoma. CNS Oncol..

[35] Hossain J.A., Latif M.A., Ystaas L.A.R., Ninzima S., Riecken K., Muller A., Azuaje F., Joseph J.V., Talasila K.M., Ghimire J. (2019). Long-term treatment with valganciclovir improves lentiviral suicide gene therapy of glioblastoma. Neuro Oncol..

[36] Drachsler M., Kleber S., Mateos A., Volk K., Mohr N., Chen S., Cirovic B., Tuttenberg J., Gieffers C., Sykora J. (2016). CD95 maintains stem cell-like and non-classical EMT programs in primary human glioblastoma cells. Cell Death Dis..

[37] Madisen L., Zwingman T.A., Sunkin S.M., Oh S.W., Zariwala H.A., Gu H., Ng L.L., Palmiter R.D., Hawrylycz M.J., Jones A.R. (2010). A robust and high-throughput Cre reporting and characterization system for the whole mouse brain. Nat. Neurosci..

[38] Mastrella G., Hou M., Li M., Stoecklein V.M., Zdouc N., Volmar M.N.M., Miletic H., Reinhard S., Herold-Mende C.C., Kleber S. (2019). Targeting APLN/APLNR Improves Antiangiogenic Efficiency and Blunts Proinvasive Side Effects of VEGFA/VEGFR2 Blockade in Glioblastoma. Cancer Res..

[39] Frisch A., Kälin S., Monk R., Radke J., Heppner F.L., Kälin R.E. (2020). Apelin Controls Angiogenesis-Dependent Glioblastoma Growth. Int. J. Mol. Sci..

[40] Kim J., Lee I.H., Cho H.J., Park C.K., Jung Y.S., Kim Y., Nam S.H., Kim B.S., Johnson M.D., Kong D.S. (2015). Spatiotemporal Evolution of the Primary Glioblastoma Genome. Cancer Cell.

[41] Chamberlain M.C. (2011). Radiographic patterns of relapse in glioblastoma. J. Neuro Oncol..

[42] Di L., Heath R.N., Shah A.H., Sanjurjo A.D., Eichberg D.G., Luther E.M., de la Fuente M.I., Komotar R.J., Ivan M.E. (2020). Resection versus biopsy in the treatment of multifocal glioblastoma: A weighted survival analysis. J. Neuro Oncol..

[43] Wick W., Stupp R., Beule A.C., Bromberg J., Wick A., Ernemann U., Platten M., Marosi C., Mason W.P., van den Bent M. (2008). A novel tool to analyze MRI recurrence patterns in glioblastoma. Neuro Oncol..

[44] Pombo Antunes A.R., Scheyltjens I., Duerinck J., Neyns B., Movahedi K., Van Ginderachter J.A. (2020). Understanding the glioblastoma immune microenvironment as basis for the development of new immunotherapeutic strategies. Elife.

[45] Pombo Antunes A.R., Scheyltjens I., Lodi F., Messiaen J., Antoranz A., Duerinck J., Kancheva D., Martens L., De Vlaminck K., Van Hove H. (2021). Single-cell profiling of myeloid cells in glioblastoma across species and disease stage reveals macrophage competition and specialization. Nat. Neurosci..

[46] Parkhurst C.N., Yang G., Ninan I., Savas J.N., Yates J.R., Lafaille J.J., Hempstead B.L., Littman D.R., Gan W.B. (2013). Microglia promote learning-dependent synapse formation through brain-derived neurotrophic factor. Cell.

[47] Volmar M.N.M., Cheng J., Alenezi H., Richter S., Haug A., Hassan Z., Goldberg M., Li Y., Hou M., Herold-Mende C. (2021). Cannabidiol converts NFkappaB into a tumor suppressor in glioblastoma with defined antioxidative properties. Neuro Oncol..

[48] Cuddapah V.A., Robel S., Watkins S., Sontheimer H. (2014). A neurocentric perspective on glioma invasion. Nat. Rev. Neurosci..

[49] Friedl P., Alexander S. (2011). Cancer invasion and the microenvironment: Plasticity and reciprocity. Cell.

[50] Gerlinger M., Rowan A.J., Horswell S., Math M., Larkin J., Endesfelder D., Gronroos E., Martinez P., Matthews N., Stewart A. (2012). Intratumor heterogeneity and branched evolution revealed by multiregion sequencing. N. Engl. J. Med..

[51] Qazi M.A., Vora P., Venugopal C., Sidhu S.S., Moffat J., Swanton C., Singh S.K. (2017). Intratumoral heterogeneity: Pathways to treatment resistance and relapse in human glioblastoma. Ann. Oncol..

[52] Woodroffe R.W., Zanaty M., Soni N., Mott S.L., Helland L.C., Pasha A., Maley J., Dhungana N., Jones K.A., Monga V. (2020). Survival after reoperation for recurrent glioblastoma. J. Clin. Neurosci..

[53] Kioi M., Vogel H., Schultz G., Hoffman R.M., Harsh G.R., Brown J.M. (2010). Inhibition of vasculogenesis, but not angiogenesis, prevents the recurrence of glioblastoma after irradiation in mice. J. Clin. Investig..

[54] Aizer A.A., Ancukiewicz M., Nguyen P.L., Shih H.A., Loeffler J.S., Oh K.S. (2014). Underutilization of radiation therapy in patients with glioblastoma: Predictive factors and outcomes. Cancer.

[55] Sulman E.P., Ismaila N., Armstrong T.S., Tsien C., Batchelor T.T., Cloughesy T., Galanis E., Gilbert M., Gondi V., Lovely M. (2017). Radiation Therapy for Glioblastoma: American Society of Clinical Oncology Clinical Practice Guideline Endorsement of the American Society for Radiation Oncology Guideline. J. Clin. Oncol. Off. J. Am. Soc. Clin. Oncol..

[56] Hossain J.A., Ystaas L.R., Mrdalj J., Valk K., Riecken K., Fehse B., Bjerkvig R., Gronli J., Miletic H. (2016). Lentiviral HSV-Tk.007-mediated suicide gene therapy is not toxic for normal brain cells. J. Gene Med..

[57] Grathwohl S.A., Kälin R.E., Bolmont T., Prokop S., Winkelmann G., Kaeser S.A., Odenthal J., Radde R., Eldh T., Gandy S. (2009). Formation and maintenance of Alzheimer’s disease beta-amyloid plaques in the absence of microglia. Nat. Neurosci..

[58] Huszthy P.C., Giroglou T., Tsinkalovsky O., Euskirchen P., Skaftnesmo K.O., Bjerkvig R., von Laer D., Miletic H. (2009). Remission of invasive, cancer stem-like glioblastoma xenografts using lentiviral vector-mediated suicide gene therapy. PLoS ONE.

[59] Le Reste P.J., Pineau R., Voutetakis K., Samal J., Jegou G., Lhomond S., Gorman A.M., Samali A., Patterson J.B., Zeng Q. (2020). Local intracerebral inhibition of IRE1 by MKC8866 sensitizes glioblastoma to irradiation/chemotherapy in vivo. Cancer Lett..

