# Supplementary material for: Comparing Tumor Cell Invasion and Myeloid Cell Composition in Compatible Primary and Relapsing Glioblastoma
Source: Cancers (Basel). 2021 Jul 20;13(14):3636. doi: 10.3390/cancers13143636 (PMC8303884; doi:10.3390/cancers13143636)
Supplement: Supplementary file 1 [file cancers-13-03636-s001.zip › cancers-1277005-supplementary.pdf]

Supplementary Materials

Comparing Tumor Cell Invasion and Myeloid Cell Composition in Compatible Primary and Relapsing Glioblastoma

Dongxu Zhao, Huabin Zhang, Ramazan Uyar, Jubayer A. Hossain, Hrvoje Miletic, Jörg-Christian Tonn, Rainer Glass and Roland E. Kälin

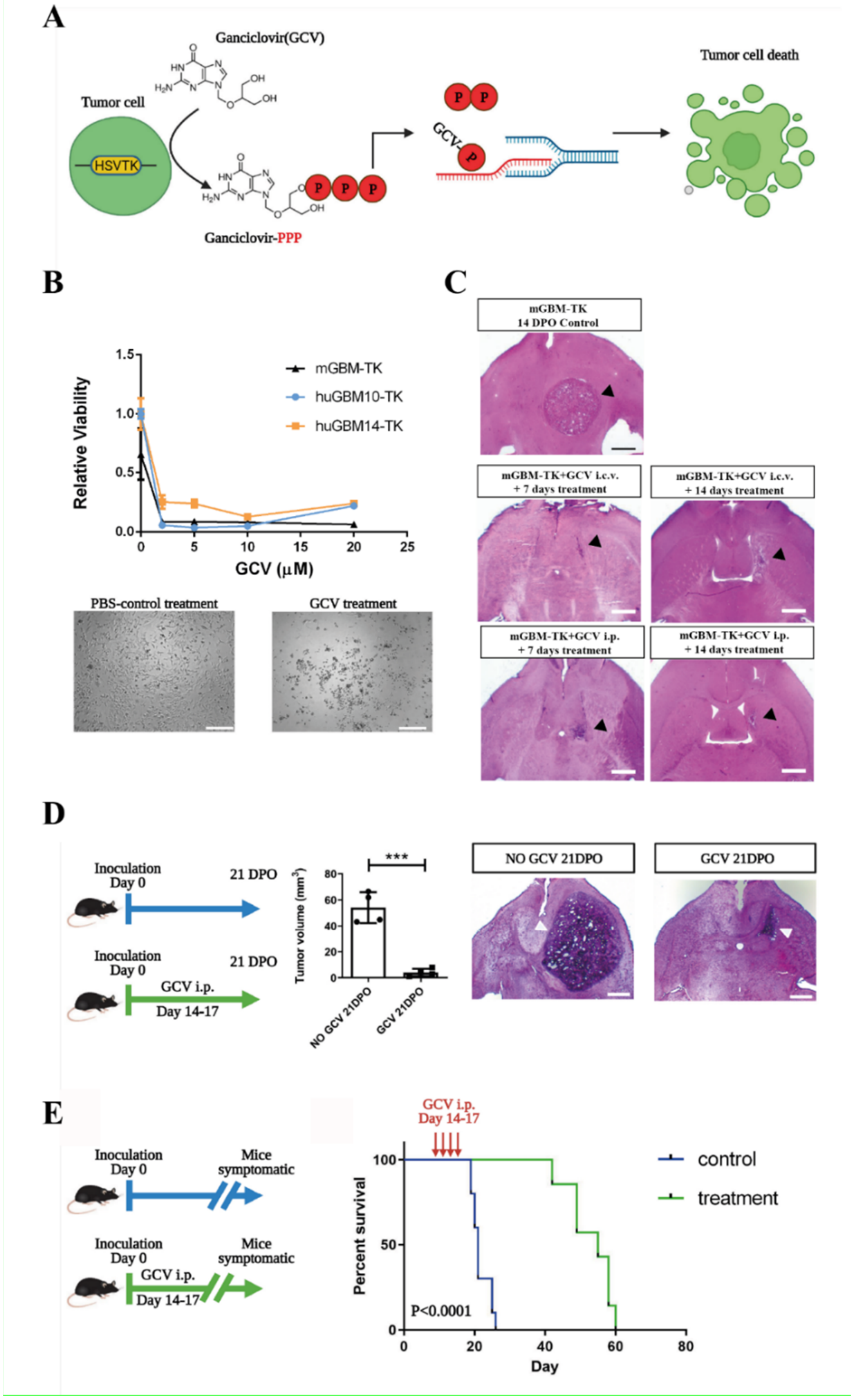

Figure S1. Murine GBM cells carrying the HSVTK suicide gene (mGBM-TK) cells) are efficiently killed by ganciclovir (GCV) treatment leading to prolonged survival of orthotopically implanted mice. (**A**)

HSVTK expression in the glioma cell line GL261 induces cells to phosphorylate the thymidine analog GCV, which results in the integration of GCV into the DNA upon replication leading to cell death. (**B**) Viability of GBM cells stably transduced with HSVTK lentivirus (murine mGBM-TK and the two human primary GBM-TK lines GBM10-TK and GBM14-TK) were assessed in a GCV dose–response curve using 2–20 μM GCV. The absorbance signal of treated cells was normalized to untreated cells and shows high lethality starting already at 2 μM range. $n = 5$ per group. Representative micrographs of cells grown under 1× PBS-control or GCV-treatment are shown. Cells grew well in the control group and died after GCV treatment. (**C**) To assess the route and dose efficient for in vivo GCV application, mGBM-TK cells were implanted and the control group analyzed at 14DPO. In the four treatment groups, GCV was applied directly into the brain by intracerebroventricular (i.c.v.;2 mM via micro-osmotic pumps cat. 1002; Alzet; Charles River) or intraperitoneal (i.p.; 25 mg/kg/day) administration for 7 or 14 consecutive days. Representative micrographs of H&E stainings show that all 4 treatment paradigms efficiently reduced the tumor mass (arrowhead). $n = 3$ per group. (**D**) Experimental schedule for orthotopic implantation of mGBM-TK cells and GCV-treatment. GCV was i.p.-injected at day 14, 15, 16 and 17 post operation (DPO) in the GCV-treatment group and all mice were sacrificed at 21 DPO. Tumor size was quantified on H&E-stained sections in $n = 4$ mice per group. Tumor volume was massively decreased in the GCV-treated group (GBM-TK + GCV) compared to the untreated controls (GBM-TK) with high significance. Representative micrographs of intracerebral tumors (arrowheads) after H&E staining are shown on the right. (**E**) Experimental schedule for the survival study. Mice were treated with GCV on day 14, 15, 16 and 17 after orthotopic mGBM-TK cell implantation or left untreated. All mice were sacrificed at humane endpoints. Survival curve of GBM-TK cell-implanted mice with (treatment group; $n = 7$) or without GCV treatment (control group; $n = 10$). The mice of the treatment group lived 2.76× longer than the mice of the untreated control group. Statistical significances were calculated using *t*-test (**D**) or a Log-rank (Mantel-Cox) test (**E**), *** $p < 0.005$. Each dot in the diagram (**D**) represents the statistical value obtained from one mouse. Scale bars represent 400 μm (**B**), 1 mm (**C**,**D**).

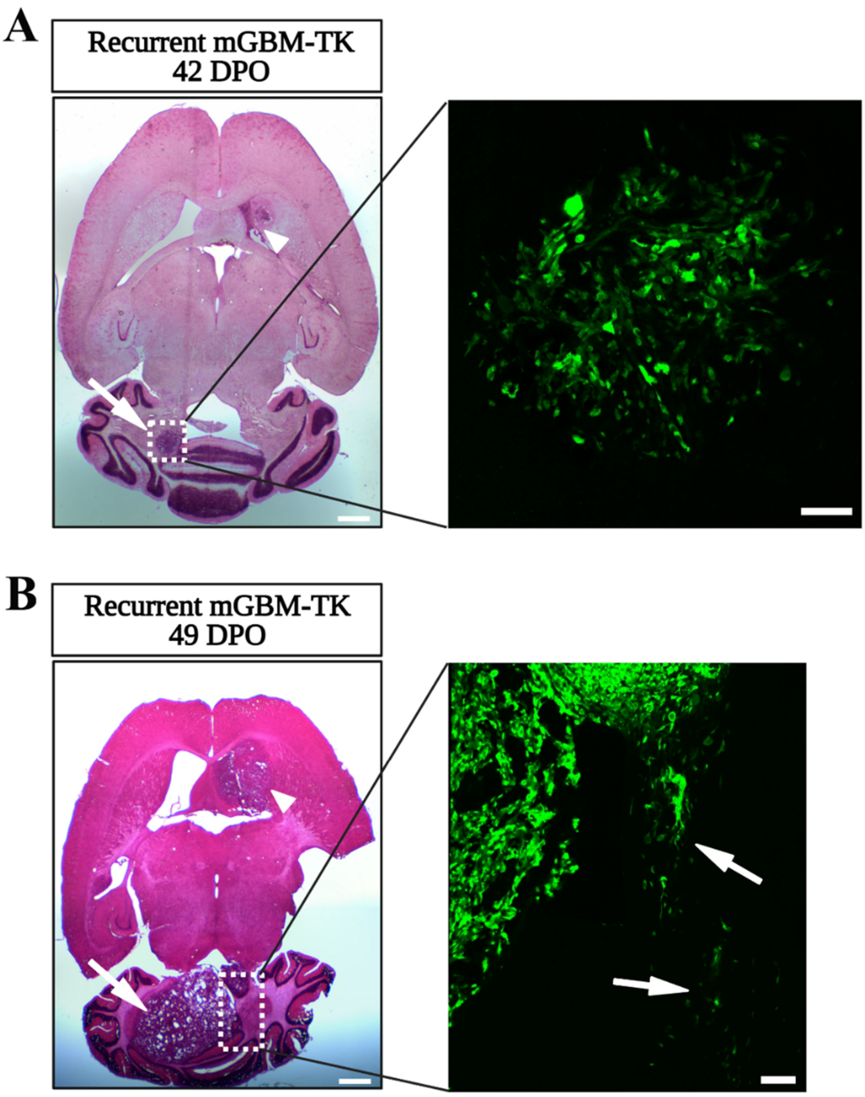

Figure S2. Tumor recurrence is found throughout the brain in local and distant sites. (**A,B**) H&E staining was performed and distant cell dense tumors (circled by dashed lines) were verified by immunofluorescent (IF) staining against GFP-positive mGBM-TK cells. Representative images show examples of local (arrowheads) and distant recurrence (arrows). Scale bars represent 1 mm (**A,B**; H&E images), 100 μm (**A,B**; IF images).

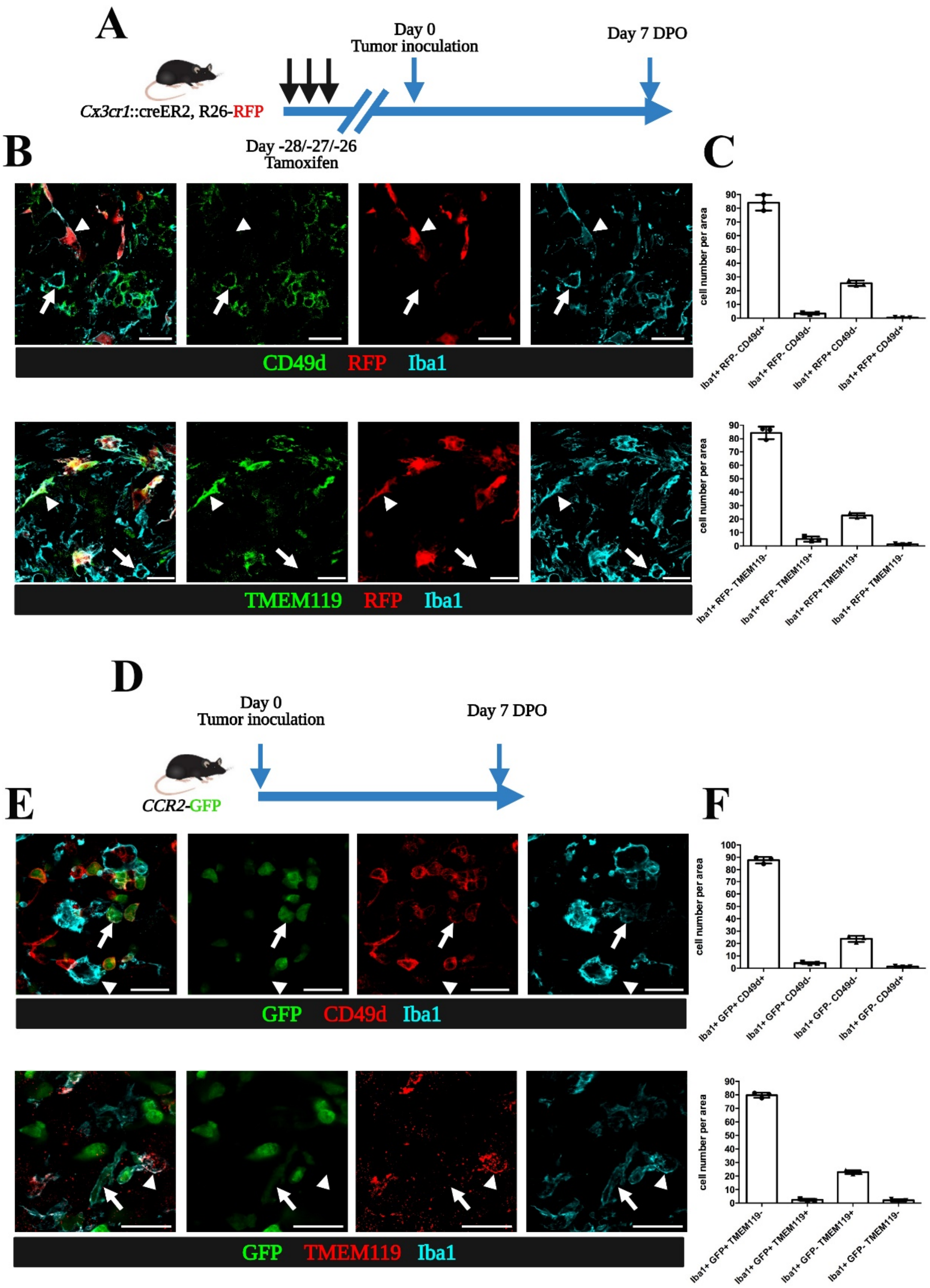

Figure S3. Transmembrane Protein 119 (TMEM119) and CD49d (encoded by integrin subunit alpha 4; ITGA4) specifically distinguish tumor-associated microglia from monocyte derived-macrophages (MDM) in mouse GBM. (**A**) Scheme for the orthotopic implantation of GL261 cells into the *Cx3cr1*::creER, R26-RFP mouse model with a TAM pulse-chase protocol to trace brain resident microglia at 7DPO. (**B**) Representative immunofluorescence images of traced microglia (red) stained for Ionized Calcium-Binding Adapter Molecule 1 (Iba1; cyan) in combination with CD49d (green) or TMEM119 (green). In the upper panel, the arrowhead indicates a traced RFP$^+$Iba1$^+$ double-positive microglia that is negative for CD49d, while RFP$^-$Iba1$^+$CD49d$^+$ MDM can be observed (arrow). The lower panel shows a traced RFP$^+$Iba1$^+$ microglia in its typical elongated morphology that is co-stained for TMEM119 (arrowhead) while RFP$^-$Iba1$^+$ cells are negative for TMEM119 (arrow). (**C**)

Quantification of the number of MDM ($Iba1^+RFP^-CD49d^+$ or $Iba1^+RFP^-TMEM119^-$) and tumor-associated microglia ($Iba1^+RFP^+CD49d^-$ or $Iba1^+RFP^+TMEM119^+$) shows that nearly all traced cells in the microglia-reporter mouse model are CD49 negative but TMEM119 positive. Number of animals per group are $n = 3$. (**D**) Scheme for orthotopic implantation of GL261 cells into the CCR2-GFP mouse model to trace peripherally invading macrophages at 7 DPO. (**E**) Representative immunofluorescence images of GFP-traced MDM (green) stained for Iba1 (cyan) in combination with CD49d (red) or TMEM119 (red). In the upper panel, the arrow indicates a GFP-traced Iba1-positive invading macrophage that is positive for CD49d, while $GFP^-Iba1^+CD49d^-$ tumor-associated microglia can be observed (arrowhead). The lower panel shows a GFP-traced Iba1-positive macrophage that is negative for TMEM119 (arrow) while GFP^-Iba1^+ cells are positive for TMEM119 (arrowhead). (**F**) Quantification of the number of MDM ($Iba1^+GFP^+CD49d^+$ or $Iba1^+GFP^+TMEM119^-$) and microglia ($Iba1^+GFP^-CD49^-$ or $Iba1^+GFP^-TMEM119^+$) shows that virtually all traced cells are CD49 positive but TMEM119 negative. Number of animals per group are $n = 3$. Abbreviations: GFP, green fluorescent protein; RFP, red fluorescent protein.

1. Supplemental Method: Viability Assay

Non-Radioactive Cell Viability/Proliferation Assay was purchased from Promega (Cat #: G4100) and applied based on manufacturer's instructions. Briefly: 7000 cells were plated in each well of a 96-well plate (5 replicates for each condition and for each cell line used), either with medium containing ganciclovir or PBS for 72 hours. On day 3, dye solution from the viability assay was added, 15 μL in each well and the plates were incubated at 37 °C under 5% CO_2/95% humidified air for 4 hours. Then, stop/dissociation solution of 100 μL was added in each well to stop the assay, disrupt the cells and dissolve the purple oxalate crystals formed within the cells to produce a colored solution. The plates are then incubated again in the same incubation conditions as before for 1 hour. The colorimetric absorbance was measured by an Elisa plate reader. The background-subtracted absorbance values were analyzed using Nalimov's Test in order to determine the outliers. The absorbance of the control was averaged (from 5 replicates) and the average value was arbitrarily set as 1. The same averaging was performed for experimental conditions and they were normalized to the average of control cells.

© 2021 by the authors. Licensee MDPI, Basel, Switzerland. This article is an open access article distributed under the terms and conditions of the Creative Commons Attribution (CC BY) license (http://creativecommons.org/licenses/by/4.0/).
